# Acetyl-L-Carnitine in the Treatment of Peripheral Neuropathic Pain: A Systematic Review and Meta-Analysis of Randomized Controlled Trials

**DOI:** 10.1371/journal.pone.0119479

**Published:** 2015-03-09

**Authors:** Sheyu Li, Qianrui Li, Yun Li, Ling Li, Haoming Tian, Xin Sun

**Affiliations:** 1 Department of Endocrinology and Metabolism, West China Hospital, Sichuan University, Chengdu, 610041, China; 2 Chinese Evidence-Based Medicine Center, West China Hospital, Sichuan University, Chengdu, 610041, China; University of Ottawa, CANADA

## Abstract

**Objective:**

Acetyl-L-carnitine (ALC), a constructive molecule in fatty acid metabolism, is an agent potentially effective for treating peripheral neuropathic pain (PNP). Its effect, however, remains uncertain. We aimed to access the efficacy and safety of ALC for the treatment of patients with PNP.

**Methods:**

We searched MEDLINE (1996–2014), EMBase (1974–2014), and CENTRAL (May 2014) up to June 27, 2014 for randomized controlled trials (RCTs) comparing ALC with placebo or other active medications in diabetic and non-diabetic PNP patients that reported the change of pain using visual analogue scale (VAS). Mean difference (MD) and 95% confidence interval (CI) were used for pooling continuous data.

**Results:**

Four RCTs comparing ALC with placebo and reporting in three articles (n = 523) were included. Compared with placebo, ALC significantly reduced VAS scores of PNP patients (MD of VAS, 1.20; 95% CI, 0.68-1.72, P <0.00001). In the subgroup analysis, the effect of ALC on VAS was similar in different administration routes (intramuscular-oral sequential subgroup: MD, 1.19; 95% CI, 0.34-2.04, P = 0.006; oral only subgroup: pooled MD, 1.15; 95%CI, 0.33-1.96, P = 0.006), and ALC appeared more effective in diabetic PNP patients than non-diabetic PNP patients (diabetic subgroup: MD, 1.47; 95%CI, 1.06-1.87, P <0.00001; non-diabetic subgroup: MD, 0.71; 95% CI, -0.01-1.43, P = 0.05). No severe adverse events were reported related to ALC. The common adverse events were pain, headache, paraesthesia, hyperesthesia, retching, biliary colic, and gastrointestinal disorders. The rates of total adverse events were similar in ALC and control group.

**Conclusion:**

The current evidence suggests that ALC has a moderate effect in reducing pain measured on VAS in PNP patients with acceptable safety. Larger trials with longer follow-up, however, are warranted to establish the effects.

## Introduction

Peripheral neuropathic pain (PNP) leads to an unpleasant experience of patients due to lesion of peripheral nerves, which may be a result of a complication of diabetes mellitus, drug adverse effect, or other origins. Although not always life-threatening, PNP substantially influences patient quality of life. PNP is associated with high prevalence of depression [1] and other psychotic disorders, which may accelerate the underlying disease.

Symptomatic treatment represents a currently primary strategy of treating PNP [2]. Despite a high cost of medication and potential adverse effects, treatment outcomes remain poor in many patients [3]. Exploring new agents for PNP is thus compelling.

Acetyl-L-carnitine (ALC) is a fundamental compound participating in the metabolism of fatty acid in mitochondria and in the modulation of nerve growth factors and neurotransmitters in the nervous system [4]. Although attempts have been made in recent years to apply ALC to the treatment of diabetic and non-diabetic peripheral neuropathy, the effects of ALC remain controversial. Therefore, we conducted a systematic review of randomized controlled trials (RCTs) to evaluate the efficacy and safety of ALC compared with placebo or other active medications in treating diabetic and non-diabetic PNP.

## Materials and Methods

We reported this study in accordance with the preferred reporting items for systematic reviews and meta-analysis (PRISMA) checklist.

### Literature search

We searched MEDLINE (1996–2014), EMBase (1974–2014), and CENTRAL (May 2014) for relevant articles till June 27, 2014, using the combinations of the following keywords: “carnitine”, “neuro”, “neuropathic pain”, and “neuropathy”. We also manually screened references of included trials for additional potential eligible studies. We checked studies for duplicate publications. We also checked if different studies used an overlapping publication; patient populations were considered overlapping when the following criteria were identical: hospital, author, study period, and intervention. For studies of duplicate or overlapping patient populations, data from the most informative or most recent publication were included in our meta-analysis.

### Inclusion and exclusion criteria

Eligible studies should meet the following inclusion criteria: (1) RCTs; (2) diabetic or non-diabetic patients with PNP diagnosed via clinical manifestations and/or neurophysiological changes in extremities; (3) ALC given as intervention, regardless of administration route; (4) placebo or other positive control drugs given as control; (5) Visual Analogue Scale (VAS) measured as the primary endpoint, while adverse events as secondary endpoints. Only studies published in English were included.

We excluded conference abstracts without original data; studies lacking adequate data for analyzing endpoints of interest; and duplicate data or overlapping studies.

### Data extraction

Two reviewers (S. L. and Q. L.) independently reviewed all searched studies. Disagreements were resolved through discussion with a third reviewer (H. T.). All data of included studies were collected independently, using a predefined form. The following data were extracted from each study: first author, year of publication, title, study design, funding source, country, gender, average age, body mass index (BMI), intervention, number of patients in treatment group and control group, length of follow-up, criteria of neuropathy diagnosis; VAS scores and number of adverse events in each group.

### Assessment of Risk of bias

Two reviewers (S. L. and Q. L.) independently assessed risk of bias of each included study using the “risk of bias” tool by the Cochrane Collaboration [5]. This tool has seven domains of bias, including generation of randomization sequence, allocation concealment, blinding of participants and care givers, blinding of outcome assessors, incomplete outcome data, selective outcome reporting, and “other issues”. The tool is accompanied with explicit and clear instructions to help assess risk of bias as “high”, “low”, or “unclear”. Reviewers addressed discrepancies of assessment through discussion.

### Statistical analysis

All the statistical analysis was performed by Review Manager 5.2.7 (Copenhagen: The Nordic Cochrane Centre, The Cochrane Collaboration, 2012). Mean difference (MD) and 95% CI were used to describe continuous data for each study. We assessed the heterogeneity among studies initially by graphically examining forest plots, and subsequently by statistical evaluation using a Chi-square test of homogeneity and evaluation of the inconsistency index (I^2^) statistic [6]. A P-value <0.1 or I^2^ >50% indicates significant statistical heterogeneity among studies. We pooled the studies using random-effects model [7] in the presence of statistical or other heterogeneity, and fixed-effects models otherwise [8]. We conducted subgroup analyses by the cause of the neuropathy (diabetes mellitus or others) and route of administration. Sensitivity analysis was performed to assess the stability of the results by removing a single trial at a time.

## Results

### Research results

**Fig 1:**
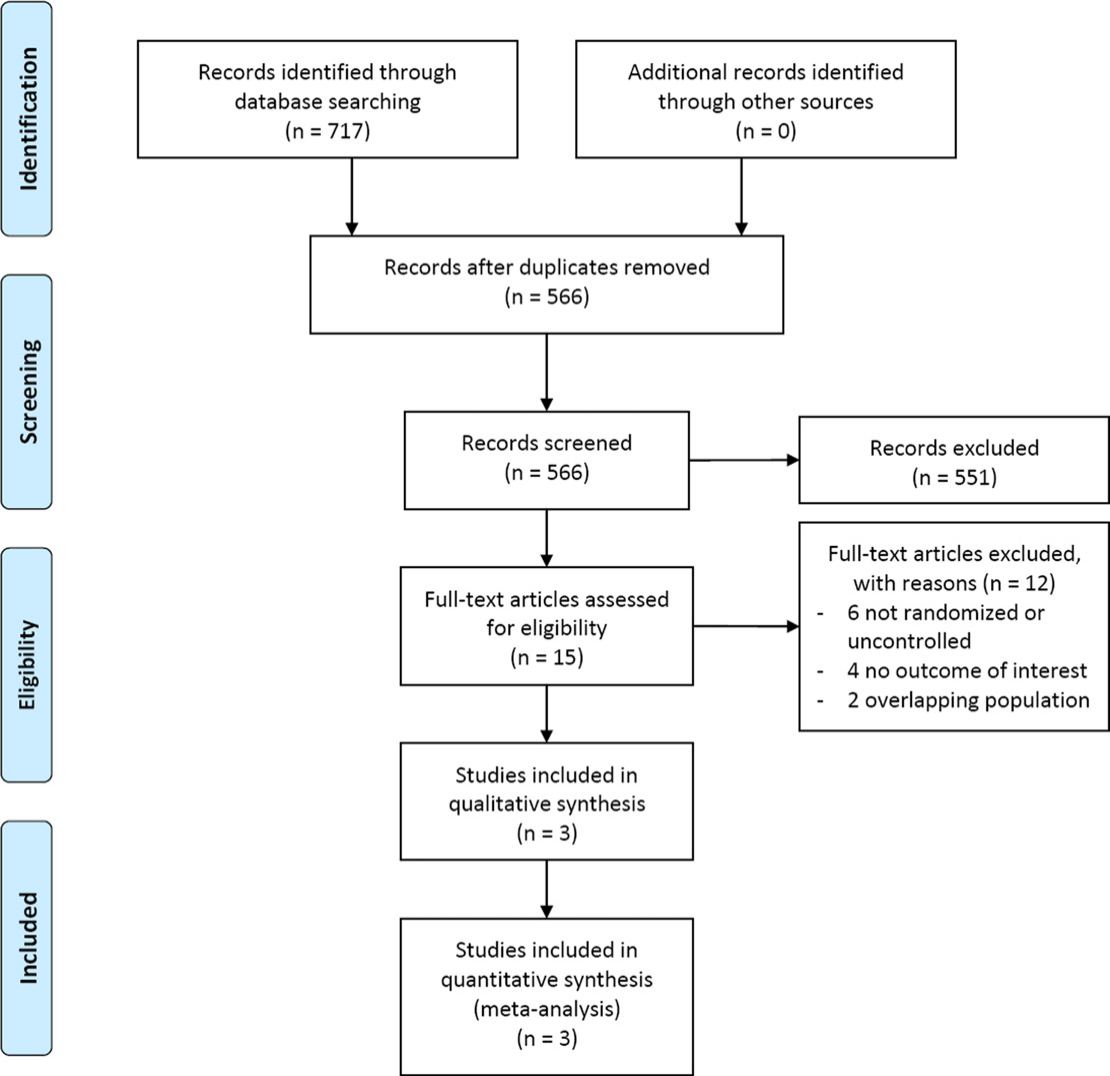
PRISMA Flow Diagram of the Meta-analysis.

Fig. 1 presents the results of article selection. After screening 717 articles, three papers [9–11] reporting four RCTs that involved 523 patients were proved eligible. Among those four RCTs, three enrolled patients with diabetic peripheral neuropathy [9,10], and one with peripheral neuropathy caused by antiretroviral agents [11]. Only one trial [11] reported information regarding age and BMI (44.4 years and 23.88 kg/m^2^ in mean, Table 1). All trials compared ALC versus placebo; two trials [9] administered ALC orally, and the other two [10,11] administered sequentially through intramuscular and oral route. The length of follow-up ranged from 14 days to one year.

**Table 1:** Characteristics of studies included in this meta-analysis.

Study ID	Design	Country	Population	Age (years)	Female (%)	BMI (kg/m^2^)	Intervention	Control	Number of patients	Time of follow-up	Funding
Sima, UC 2005 [9]	RCT	USA, Canada	DPN	NR	NR	NR	3000mg/d, p.o.	Placebo	118	52 weeks	NR
Sima, UCE 2005 [9]	RCT	USA, Canada, Europe	DPN	NR	NR	NR	3000mg/d, p.o.	Placebo	119	52 weeks	NR
De Grandis, 2002 [10]	RCT	Italy	DPN	NR	43.5	NR	1000mg/d, i.m., for 10days;then 2000mg/d, p.o. for 355days	Placebo	147	1 year	Sigma-Tau, Italy
Youle, 2007 [11]	RCT	Argentina, Israel, Italy, the Netherlands and the UK	PN caused by ATV	44.4	20.0	23.88	1000mg/d i.m. for 14days;then 2000mg/d p.o.	Placebo	87	14 days	Sigma-Tau, Italy

BMI = body mass index; RCT = randomized controlled trial; DPN = diabetic peripheral neuropathy; PN = peripheral neuropathy; ATV = antiretroviral agent; NR = not reported

Quality assessment result is shown in S1 Fig. and S2 Fig. Only one trial [10] was free of all biases except reporting bias. Other trials [9,11] were unclear in selection bias, performance bias, detection bias, and selective bias.

### Effect of ALC on pain

**Fig 2:**
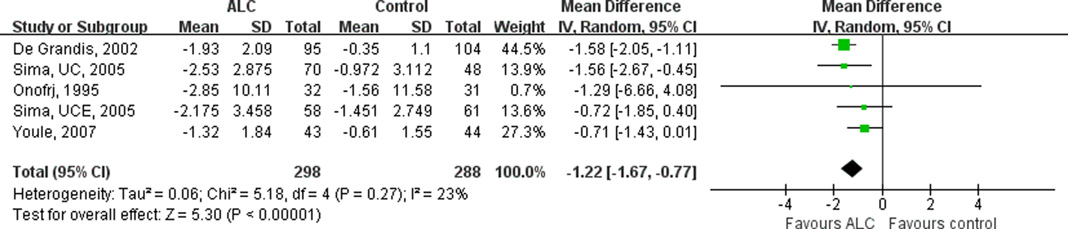
Overall Meta-analysis on the VAS Scores. Patients receiving ALC showed significantly more reduction in VAS scores than those receiving placebo. The values presented referred to the change of VAS scores from baseline. VAS = Visual Analogue Scale; ALC = acetyl-l-carnitine; UCE = U.S.-Canadian-European Study; UC = U.S.-Canadian Study; SD = standard deviation; CI = confidence interval.

All the 4 trials [9–11] reported the effect of ALC on pain measured with VAS scores. Although no significant statistical heterogeneity was present among the studies (I^2^ = 42%, P = 0.16), random-effects model was used in the analysis due to different participants. The pooled results showed that ALC slightly reduced pain compared with placebo with statistical significance (MD of VAS, 1.20; 95%CI, 0.68–1.72, P < 0.00001, Fig. 2).

**Fig 3:**
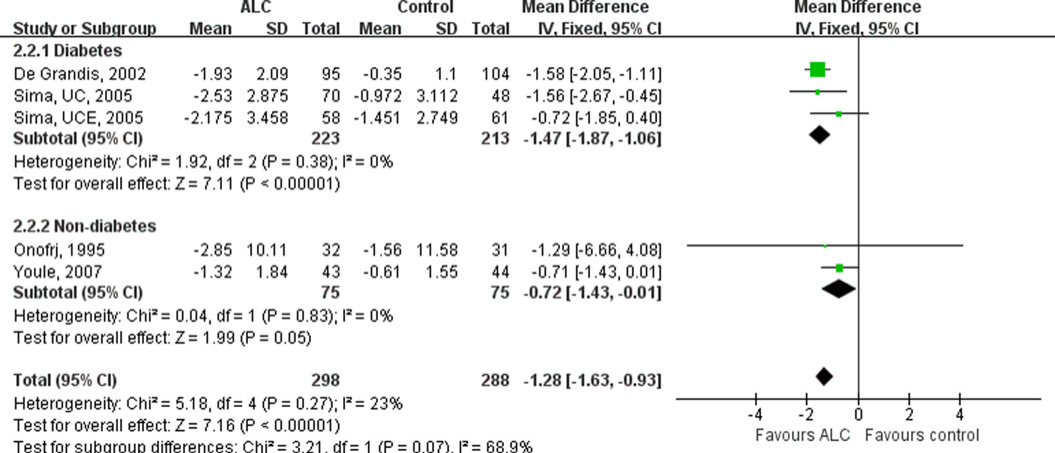
Subgroup-analysis on the VAS Scores of the Diabetic and Non-diabetic Patients. Subgroup-analysis was performed by subdividing RCTs according to whether the peripheral neuropathy diagnosed in patients was diabetic or non-diabetic. Taking ALC decreased VAS scores significantly in diabetic patients. VAS = Visual Analogue Scale; ALC = acetyl-l-carnitine; UCE = U.S.-Canadian-European Study; UC = U.S.-Canadian Study; SD = standard deviation; CI = confidence interval.

Subgroup analysis was introduced by subdividing RCTs according to whether the peripheral neuropathy diagnosed in patients was diabetic or non-diabetic. Three RCTs [9,10] enrolled patients with DPN, while one [11] enrolled patients with non-diabetic PNP. No significant heterogeneity was found among RCTs in the diabetic subgroup (I^2^ = 0%, P = 0.38), and thus fix-effects model was chosen. The result (Fig. 3) showed that, in the diabetic subgroup, patients receiving ALC presented a significant decreased in VAS scores compared with those receiving placebo (MD, 1.47; 95% CI, 1.06–1.87, P <0.00001). A less significant reduction of VAS scoring was observed in the only report of non-diabetic group (MD, 0.71; 95% CI, -0.01–1.43, P = 0.05).

**Fig 4:**
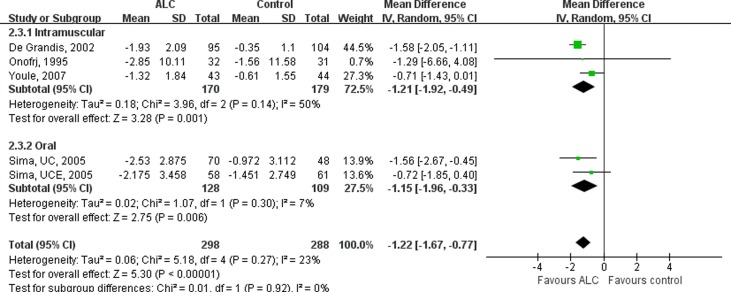
Subgroup-analysis on the VAS Scores by Subdividing RCTs according to the Route of Administration. Oral administration of ALC decreased VAS scores significantly. VAS = Visual Analogue Scale; ALC = acetyl-l-carnitine; UCE = U.S.-Canadian-European Study; UC = U.S.-Canadian Study; SD = standard deviation; CI = confidence interval.

We also conducted a subgroup analysis according to the route of ALC administration. Two RCTs [10,11] administered ALC sequentially by intramuscular and oral routes, and two other RCTs [9] orally only. The effects on VAS scores were similar between the two subgroups: oral administration subgroup (MD, 1.15; 95%CI, 0.33–1.96, P = 0.006) and sequential administration subgroup (MD, 1.19; 95% CI, 0.34–2.04, P = 0.006) (Fig. 4).

### Adverse events

There was no statistically significant difference in the incidence of adverse events between treatment groups (Table 2).

**Table 2:** Adverse Events.

Author	Adverse events	Possible drug-related events	Dropout
**Sima [9]**	No increased adverse events had been reported in ALC groups of both RCTs.	Non-reported	No safety dropouts and 9 drug-unrelated deaths were reported in both RCTs.
**De Grandis [10]**	No significant difference was reported between the ALC and placebo group.	Possibly or probably drug-related adverse events included headache, facial paraesthesia, nausea, retching, biliary colic, vomiting, epigastric pain, and gastrointestinal disorders.	Six patients in the ALC group and 2 in the placebo group dropped out as a consequence of adverse events.
**Youle [11]**	Nine patients (20.9%) in the ALC group and 14 patients (29.8%) in the placebo group experienced 16 and 20 adverse events, respectively. One drug-unrelated serious adverse event was reported in either group.	Possible drug related events included paraesthesia, pain, anorexia, dry mouth, and neuropathy.	Two patients in ALC group and 6 in placebo group dropped out. Only 1 in ALC group dropped out due to adverse event.

ALC = acetyl-l-Carnitine; RCT = randomized controlled trial.

## Discussion

PNP has been emphasized on largely for its predominant unpleasant feeling, and has a strong impact on quality of life [12]. Diabetes is often considered the primary cause of peripheral neuropathy. About one third of diabetic patients suffer from painful neuropathy [13]. Although widely used for PNP, ALC is not even noted in the most current guideline for painful diabetic neuropathy [14]. To our best knowledge, there has not been a systematic review published that specifically addresses the effect of ALC for PNP. We thus have systematically collected all relevant RCTs, and our results suggested that ALC is effective and safe for alleviating pain of PNP patients.

ALC is essential in the metabolism of fatty acid in mitochondria, and could raise the pain threshold by enhancing the activity of cholinergic nerves [15], which is thought to be associated with the expression of GRM2 gene [16]. Carnitine deficiency reduced energy synthesis by impairing fatty acid degradation [17], and was reported to be associated with diabetes and its complications [18]. Patients with diabetic retinopathy, diabetic neuropathy, and both diabetes and hyperlipidemia had a significantly lower total and free carnitine level compared with the diabetic patients without complications or co-morbidities. Several observational studies indicated an obvious improvement of symptoms after supplemented with ALC in PNP patients [19–21].

The current meta-analysis indicated ALC reduce VAS with statistical significance. Although the scoring process is subjective, VAS has been established to be a novel and reliable symptom examination of PNP, an also subjective experience. It indicated patients with PNP may gain benefit from ALC administration. Another study [21] using the modified Short Form McGill Pain Questionnaire showed a significant pain reduction at week three of ALC treatment compared with baseline. Meanwhile, electromyography, another traditional examination of PNP according to the latest guideline [14], was compared between ALC and placebo group in several studies [10, 21–23], suggesting a significant improvement of both sensory and motor nerve conduction velocity after treatment of ALC. Apart from PNP, it is also reported [24, 25] that root pain could be relieved by ALC treatment.

According to the subgroup analysis, patients with diabetic neuropathic pain appeared to have a better response from ALC compared with those due to a non-diabetic cause, which is consistent with recent systematic reviews [26,27]. One paper [26] reviewing diabetic neuropathy included two RCTs [10,28] and concluded that ALC could relief neuropathic pain, while another paper reviewing HIV-associated neuropathy included one RCT [11] and suggested no superiority of ALC to placebo. Such results may be associated with restoration of carnitine level in diabetic PNP patients. Meanwhile, as only one RCT [11] on HIV-associated neuropathy was reviewed, limitations of the trial itself such as the low dose and short follow up period might also contribute to the inefficacy. Due to the limited evidence, however, a definitive conclusion is not yet available. Another subgroup analysis indicated oral administration had similar effect with intramuscular-oral sequential administration but potential better convenience of application. The effective dose of ALC could not be concluded yet. However, our included studies intervening with a dosage higher than 2000mg/d seemed to show more benefit to patients [9,10], while one subgroup [9] with 1500mg/d showed less reduction in VAS (p value not provided). Further trials evaluating different doses of ALC are necessary.

With a chemical structure similar to other essential compounds in human body, administration of carnitine was supposed to be not harmful. Our results also indicated ALC was a safe agent without additional adverse effect compared with placebo.

This meta-analysis has several limitations. First, the evidence is limited to draw a definitive conclusion: only four small or moderate sized RCTs were included, and the risk of bias is generally moderate. Second, VAS score was a subjective outcome and could be significantly affected by both patients and physicians. Third, we used mean and SDs for pooling VAS. This presents a potential limitation as it is possible that the data on VAS is not normally distributed in RCTs. Nonetheless, one [11] of our included trials has clearly stated its normal distribution of the VAS data. Additionally, published systematic reviews [29, 30] have consistently used mean and SD to analyze VAS. Forth, the length of follow-up was relatively short in those trials; the long-term impact of ALC is unknown. Fifth, most trials were performed on or led by Italian, and funded by Sigma-Tau, the manufacturer of ALC agent. Thus, a limitation of ethnicity and a potential commercial bias could not be excluded in the study.

In conclusion, the current evidence suggests that ALC seems effective and safe in the treatment of PNP, especially of diabetic PNP. Oral administration of ALC may be recommended due to its similar efficacy but easier administration. However, further trials with larger and various population and longer follow-up are needed.

## Supporting Information

S1 Fig Risk of Bias Graph. Review about each risk of bias item presented as percentages across all included studies.
(PNG)

S2 Fig Risk of Bias Summary. Review about each risk of bias item for each included study.
(PNG)

S1 Information Full-text examined articles and the reasons for exclusion. (DOC)

S1 PRISMA Checklist PRISMA Checklist for the Meta-analysis. (DOC)
