# Correction: Acetyl-L-carnitine in the Treatment of Peripheral Neuropathic Pain: A Systematic Review and Meta-analysis of Randomized Controlled Trials

**DOI:** 10.1371/journal.pone.0129991

**Published:** 2015-06-12

**Authors:** Sheyu Li, Qianrui Li, Yun Li, Ling Li, Haoming Tian, Xin Sun

In Figs 2, 3, and 4, a trial named “Onofrj, 1995” should not be included. Please see the corrected figures here.

**Fig 2:**
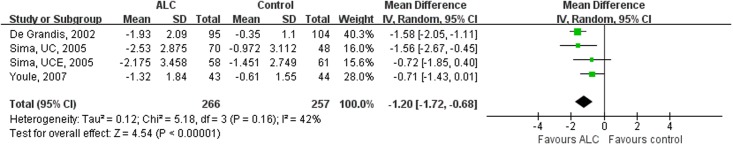
Overall Meta-analysis on the VAS Scores. Patients receiving ALC showed significantly more reduction in VAS scores than those receiving placebo. The values presented referred to the change of VAS scores from baseline. VAS = Visual Analogue Scale; ALC = acetyl-l-carnitine; UCE = U.S.-CanadianEuropean Study; UC = U.S.-Canadian Study; SD = standard deviation; CI = confidence interval.

**Fig 3:**
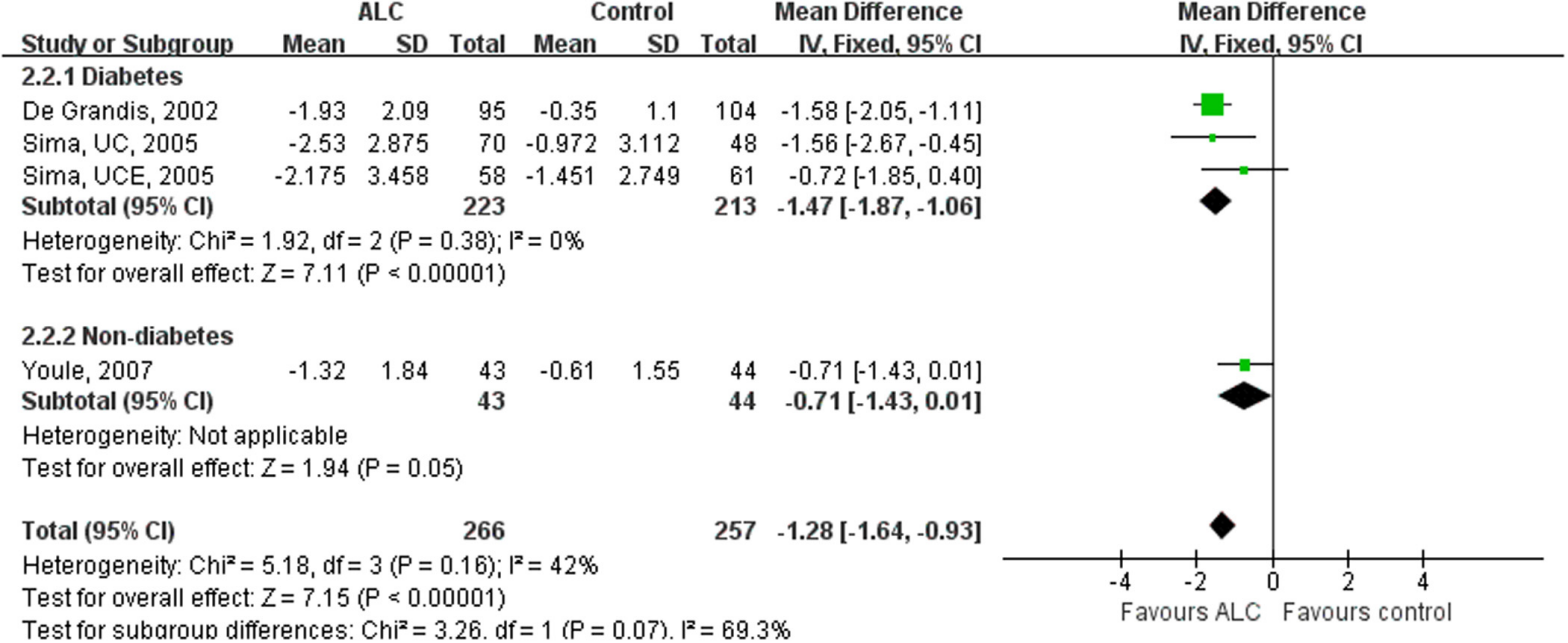
Subgroup-analysis on the VAS Scores of the Diabetic and Non-diabetic Patients. Subgroup-analysis was performed by subdividing RCTs according to whether the peripheral neuropathy diagnosed in patients was diabetic or non-diabetic. Taking ALC decreased VAS scores significantly in diabetic patients. VAS = Visual Analogue Scale; ALC = acetyl-l-carnitine; UCE = U.S.-Canadian-European Study; UC = U.S.-Canadian Study; SD = standard deviation; CI = confidence interval.

**Fig 4:**
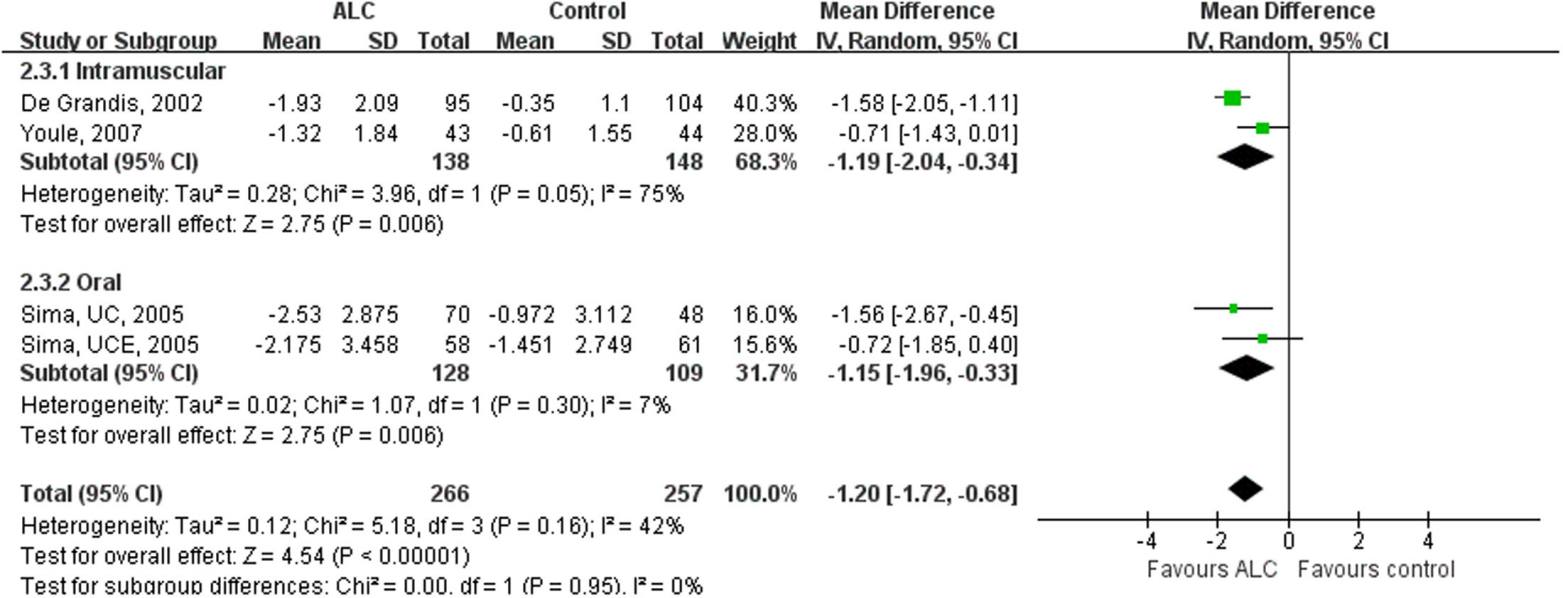
Subgroup-analysis on the VAS Scores by Subdividing RCTs according to the Route of Administration. Oral administration of ALC decreased VAS scores significantly. VAS = Visual Analogue Scale; ALC = acetyl-l-carnitine; UCE = U.S.-Canadian-European Study; UC = U.S.-Canadian Study; SD = standard deviation; CI = confidence interval.
