# Correction: Correction: Acetyl-L-carnitine in the Treatment of Peripheral Neuropathic Pain: A Systematic Review and Meta-analysis of Randomized Controlled Trials

**DOI:** 10.1371/journal.pone.0139292

**Published:** 2015-09-23

**Authors:** The PLOS ONE Staff

## Notice of Republication

This Correction was republished on September 3, 2015 to correct errors in the reference that were introduced during the typesetting process. The publisher apologizes for the errors.
