# Supplementary figures and images for: Acetyl-L-Carnitine in the Treatment of Peripheral Neuropathic Pain: A Systematic Review and Meta-Analysis of Randomized Controlled Trials
Source: PLoS One. 2015 Mar 9;10(3):e0119479. doi: 10.1371/journal.pone.0119479 (PMC4353712; doi:10.1371/journal.pone.0119479)

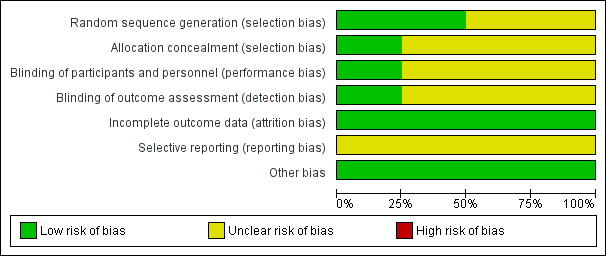

Supplement: S1 Fig — Review about each risk of bias item presented as percentages across all included studies. (PNG) [file pone.0119479.s001.png]

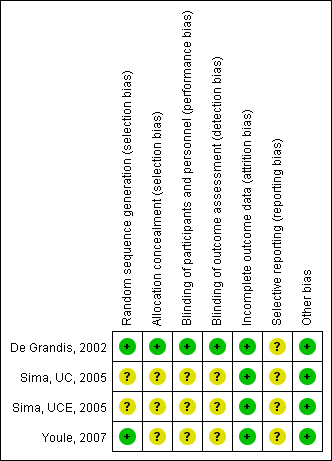

Supplement: S2 Fig — Review about each risk of bias item for each included study. (PNG) [file pone.0119479.s002.png]
