# Supplementary material for: Acetyl-L-Carnitine in the Treatment of Peripheral Neuropathic Pain: A Systematic Review and Meta-Analysis of Randomized Controlled Trials
Source: PLoS One. 2015 Mar 9;10(3):e0119479. doi: 10.1371/journal.pone.0119479 (PMC4353712; doi:10.1371/journal.pone.0119479)
Supplement: S1 Information — (DOC) [file pone.0119479.s003.doc]

**S1 Information. Full-text examined articles and the reasons for exclusion.**

After screening the full text for 16 papers, only three [1-3] met our inclusion criteria. Six papers [4-9] were excluded due to being not randomized or uncontrolled. Three papers [10-12] did not provide sufficient information about changes of VAS, and one paper [13] lacked clearly reported number of participants in the intervention and control group. Three papers [2,14,15] were from an overlapping population, defined as studies from the same hospital, with identical first author name, study period, and intervention. The start and end date of each trial was crucial but not provided by any paper. And thus only data from the most recent publication with the longest follow-up duration [2] were included in our meta-analysis.

**References**

1. Sima AA, Calvani M, Mehra M, Amato A (2005) Acetyl-L-carnitine improves pain, nerve regeneration, and vibratory perception in patients with chronic diabetic neuropathy: an analysis of two randomized placebo-controlled trials. Diabetes Care 28: 89-94.

2. De Grandis D, Minardi C (2002) Acetyl-L-carnitine (levacecarnine) in the treatment of diabetic neuropathy. A long-term, randomised, double-blind, placebo-controlled study. Drugs R D 3: 223-231.

3. Youle M, Osio M (2007) A double-blind, parallel-group, placebo-controlled, multicentre study of acetyl L-carnitine in the symptomatic treatment of antiretroviral toxic neuropathy in patients with HIV-1 infection. HIV Med 8: 241-250.

4. Bianchi G, Vitali G, Caraceni A, Ravaglia S, Capri G, et al. (2005) Symptomatic and neurophysiological responses of paclitaxel- or cisplatin-induced neuropathy to oral acetyl-L-carnitine. Eur J Cancer 41: 1746-1750.

5. Herzmann C, Johnson MA, Youle M (2005) Long-term effect of acetyl-L-carnitine for antiretroviral toxic neuropathy. HIV Clin Trials 6: 344-350.

6. Maestri A, De Pasquale Ceratti A, Cundari S, Zanna C, Cortesi E, et al. (2005) A pilot study on the effect of acetyl-L-carnitine in paclitaxel- and cisplatin-induced peripheral neuropathy. Tumori 91: 135-138.

7. Osio M, Muscia F, Zampini L, Nascimbene C, Mailland E, et al. (2006) Acetyl-l-carnitine in the treatment of painful antiretroviral toxic neuropathy in human immunodeficiency virus patients: an open label study. J Peripher Nerv Syst 11: 72-76.

8. Uzun N, Sarikaya S, Uluduz D, Aydin A (2005) Peripheric and automatic neuropathy in children with type 1 diabetes mellitus: the effect of L-carnitine treatment on the peripheral and autonomic nervous system. Electromyogr Clin Neurophysiol 45: 343-351.

9. Ulvi H, Aygul R, Demir R. (2010) Effect of L-carnitine on diabetic neuropathy and ventricular dispersion in patients with diabetes mellitus. Turk J Med Sci 40 (2): 169-175.

10. Campone M, Berton-Rigaud D, Joly-Lobbedez F, Baurain JF, Rolland F, et al. (2013) A double-blind, randomized phase II study to evaluate the safety and efficacy of acetyl-L-carnitine in the prevention of sagopilone-induced peripheral neuropathy. Oncologist 18: 1190-1191.

11. Hershman DL, Unger JM, Crew KD, Minasian LM, Awad D, et al. (2013) Randomized double-blind placebo-controlled trial of acetyl-L-carnitine for the prevention of taxane-induced neuropathy in women undergoing adjuvant breast cancer therapy. J Clin Oncol 31: 2627-2633.

12. Mondal S, Choudhury KB, Sharma S, Gupta A, Dutta S. (2014) Comparative study among glutamine, acetyl-L-carnitine, vitamin-E and methylcobalamine for treatment of paclitaxel-induced peripheral neuropathy. Clinical Cancer Investigation Journal 3: 213-219.

13. Quatraro A, Roca P, Donzella C, Acampora R, Marfella R, et al. (1995) Acetyl-L-carnitine for symptomatic diabetic neuropathy. Diabetologia 38: 123.

14. De Grandis D,Santoro L, Di Benedetto P (1995) L-acetylcarnitine in the treatment of patients with peripheral neuropathies: a short term, double-blind clinical study of 426 patients. Clin. Drug Invest 10 (6): 317-322.

15. De Grandis D (1998) Tolerability and efficacy of L-acetylcarnitine in patients with peripheral neuropathies: a short-term, open multicentre study. Clin Drug Invest. 15 (2): 73-79.
